# Two Cases of Allergic Fungal Sinusitis with Differing Postoperative Course

**DOI:** 10.1155/2019/9598283

**Published:** 2019-11-21

**Authors:** Yuma Matsumoto, Hidenori Yokoi, Michitsugu Kawada, Masachika Fujiwara, Koichiro Saito

**Affiliations:** ^1^Department of Otorhinolaryngology, Kyorin University School of Medicine, Mitaka, Japan; ^2^Department of Pathology, Kyorin University School of Medicine, Mitaka, Japan

## Abstract

Allergic fungal sinusitis (AFS) often develops in unilateral paranasal sinuses, which must be differentiated from tumors. When AFS develops on both sides, however, it must be differentiated from eosinophilic chronic sinusitis with evident eosinophilic infiltration at nasal/paranasal sinus mucosa; both conditions are highly recurrent and commonly considered intractable paranasal sinusitis. Surgical correction is the primary treatment method for AFS, as it is essential to connect the paranasal sinus communication to ensure exhaustive resection of the pathologic mucosa and for nasal steroids to reach each paranasal sinus. We recently encountered two AFS cases with differing postoperative courses. Case 1 showed evident exacerbation in the computed tomography findings, which suggests progression to eosinophilic sinusitis. Case 2 showed a benign prognosis without recurrence. Close long-term follow-up should be mandatory after surgery for the treatment of AFS.

## 1. Introduction

Allergic fungal sinusitis (AFS) is a paranasal sinus disease similar to allergic bronchopulmonary aspergillosis (ABPA) that was reported for the first time in the 1980s by Millar et al. [1, 2]. It is considered a type I or III allergic reaction to fungus at the paranasal sinus mucosa; however, a recent study reported that *Aspergillus* infiltration at the paranasal sinuses induces an atopic reaction or immune response disorder, exacerbating nasal polyps by inducing the response of T-helper 17 cells [3]. New findings regarding the pathology of the disease are expected following further investigation.

Since AFS usually develops on one side of the paranasal sinus and presents as bone thinning in computed tomography (CT) images, it often requires careful differentiation from nasal/paranasal sinus tumors [4]. Among the preoperative diagnosis indexes, the presence/absence of allergic mucin, multiple nasal polyps, or bone thinning in CT images are essential [5]; a positive reaction to the fungi-specific immunoglobulin E (IgE) using the serum or no signal readings in T2-weighted magnetic resonance imaging (MRI) are also highly supportive indexes for diagnosis. Primary treatment options include exhaustive resection of the lesion and rebuilding the nasal/paranasal sinus pathways via surgical operations [6]. AFS recurs when the fungal antigen is not sufficiently removed; most of these cases require another surgery [7]. In this paper, we report two recent AFS cases that required differentiation at initial diagnosis from nasal cancer and showed differing clinical observations in their postoperative courses. Herein, we also provide a literature review.

## 2. Case 1

**Figure 1:**
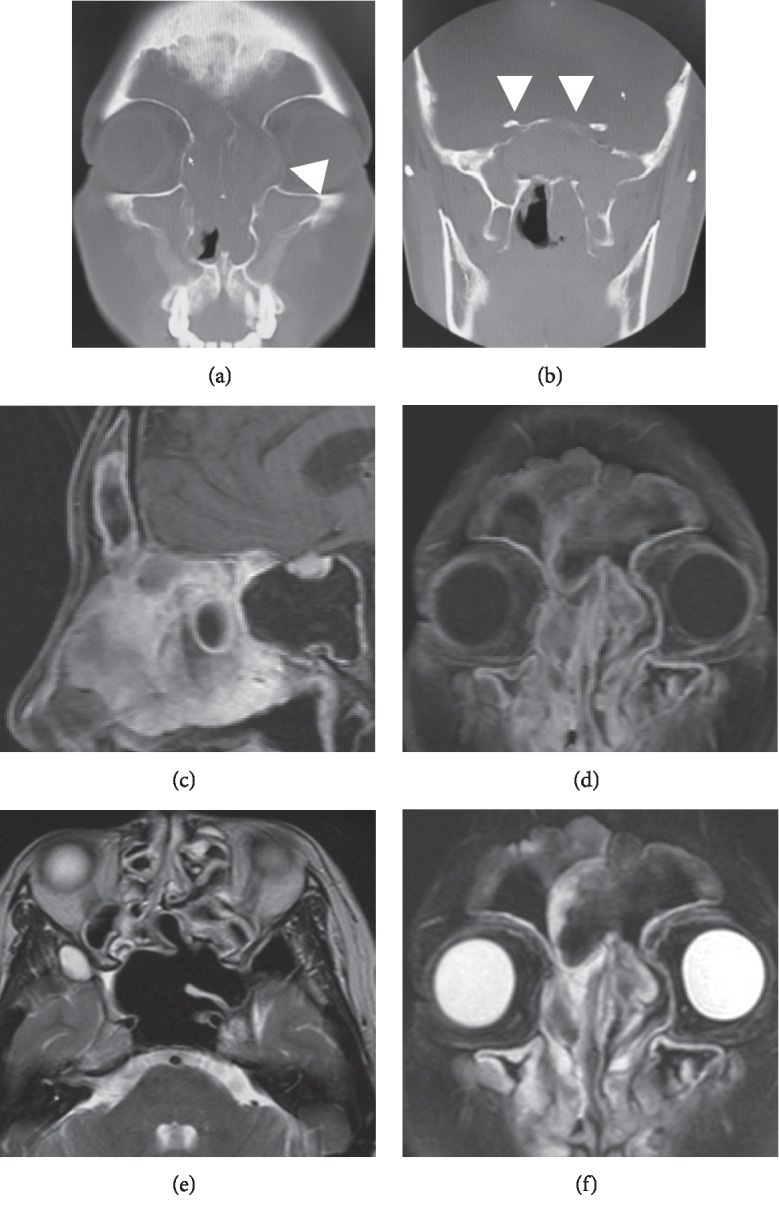
Image findings of Case 1 at the initial diagnosis. (a, b) Computed tomography images of the paranasal cavity (coronal section); bone thinning is evident (white arrow). (c) Gadolinium- (Gd-) enhanced T1-weighted MRI of the paranasal cavity (sagittal section). (d) Gd-enhanced T1-weighted image (coronal section). (e) T2-weighted image (axial section). (f) T2-weighted image (coronal section). Slightly high signals are evident at the paranasal sinus mucosa or nasal mucosa in the T1-weighted images, and a robust enhancing effect is evident in the Gd-enhanced image. Nonsignal regions are evident at the frontal, ethmoid, and sphenoid sinuses, along with an area showing low- to faint-high signal, indicating the possibility of allergic mucin in the nasal cavity. In the T2-weighted image, the paranasal sinus mucosa or nasal mucosa shows high signal levels, and most of the area with possible allergic mucin shows a low signal level.

A 40-year-old female patient had suffered from allergic rhinitis symptoms such as nasal obstruction or nasal mucus since early childhood. She visited the local ear nose and throat clinic, complaining mainly of nasal obstruction and swelling at the left internal canthus and left dacryorrhea. As CT showed soft-tissue contrast at all of the paranasal sinuses, as well as bone thinning at the middle cranial bottom, at both paries medialis orbitae and at the bottom of the sphenoidal sinus (Figures 1(a) and 1(b)), a possible tumorous lesion was undeniable. She was referred to our hospital for further diagnosis. A polypous lesion occupying both nasal cavities, yellow nasal mucus, and highly viscous colloidal mucus were evident at the initial diagnosis. As the possible tumorous lesion required a differential diagnosis, we conducted MRI scanning along with a blood test, allergy test, and the close examination of the tumor marker (SCC). An increase in the inflammatory response was not evident in the blood test, which showed the levels of eosinophil and nonspecific IgE to be high at 11.3% (598.9/*μ*L) and 9,427 IU/ml, respectively. The antigen-specific IgE testing showed *Alternaria* at class 4 and *Aspergillus* at class 1; the reading of *β*-D glucan was 7.7 pg/ml (threshold of 0–20 pg/ml), and the reading of SCC was 2.6 ng/ml (threshold of 0–1.5 ng/ml). On the T1-weighted MRI, slightly high signals were evident at the paranasal sinus mucosa and nasal mucosa; a robust enhancing effect was evident in the gadolinium- (Gd-) enhanced image. Nonsignal regions were evident at frontal, ethmoid, and sphenoid sinuses, along with the area showing low- to faint-high signals, indicating possible allergic mucin in the nasal cavity. In the T2-weighted image, the paranasal sinus mucosa or nasal mucosa showed high signal levels and most of the area of possible allergic mucin showed low signal level (Figures 1(c)–1(f)).

**Figure 2:**
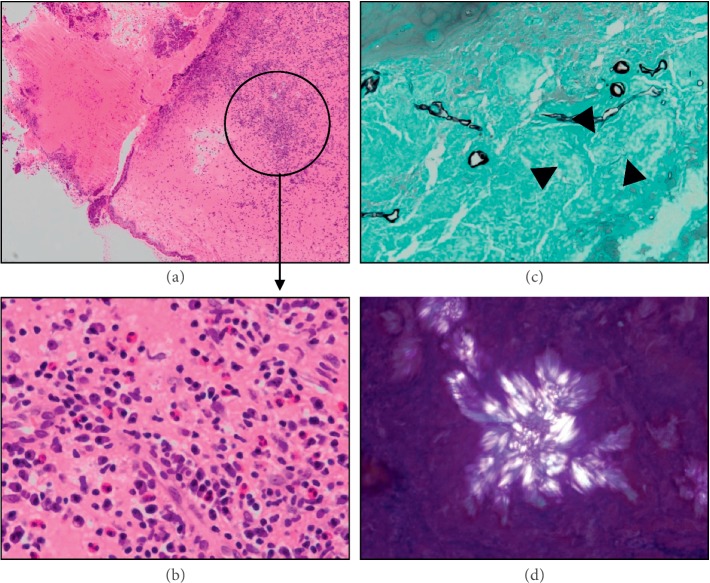
Pathological findings of Case 1. (a, b) Hematoxylin-eosin stain; eosinophilic infiltration at the nasal polyp is evident. (c) Grocott stain. Mycelia are evident in the eosinophilic mucin, but it is not possible to identify the fungus (black arrow). (d) Image from the polarizing microscope. Calcium oxalate crystals are evident in the eosinophilic mucin.

According to these results, we dismissed the possibility of a malignant tumor and suspected AFS. We therefore performed endoscopic sinus surgery. We removed nasal polyps and colloidal mucus in the nasal cavities on both sides to clear the pathways to all of the paranasal sinuses. We then completed the operation by performing a thorough irrigation and suction of the nasal/paranasal sinus with the powered Hydrodebrider Endoscopic Sinus Irrigation SystemⓇ (Hydrodebrider) (Medtronic, USA). We confirmed the infiltration of eosinophils alone in the nasal mucosa sample collected during the surgery; the presence of fungus was not evident. Also, mycelia, calcium oxalate crystals, and Charcot–Leyden crystals were evident in the colloidal mucus (Figure 2).

**Figure 3:**
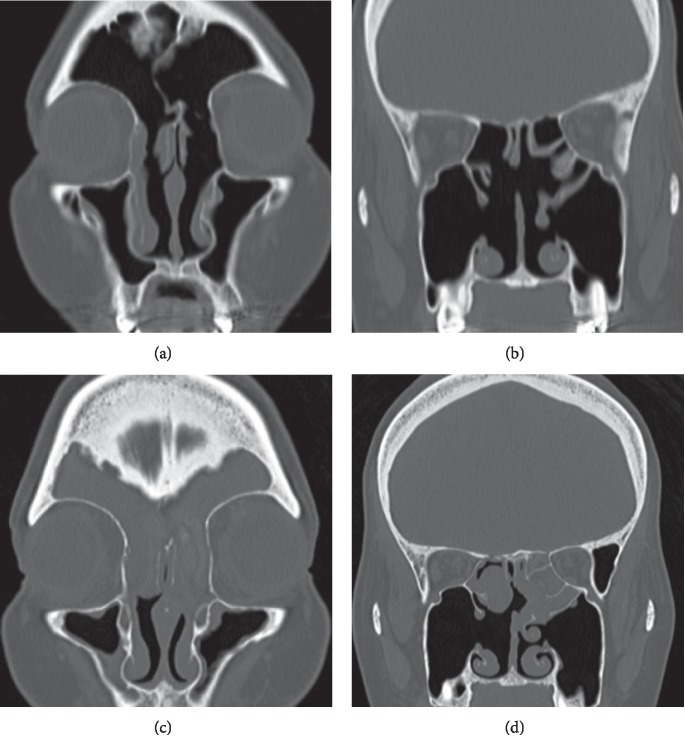
Case 1 recurrence. (a, b) Computed tomography (CT) image of the paranasal cavity in Case 1 after the first surgery. Pathways from the nasal cavity to each paranasal cavity are cleared. (c, d) CT image of the paranasal cavity in Case 1 after disease recurrence. Contrasts are evident in the ethmoid and frontal sinuses on both sides.

**Figure 4:**
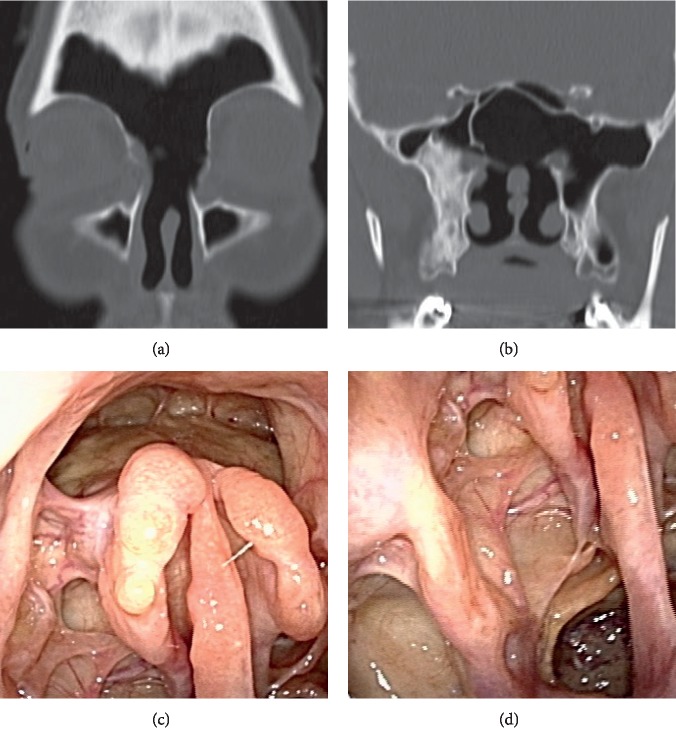
Continued remission in Case 2. (a, b) Computed tomography image findings of Case 2 after the second surgery. (c, d) Pathological findings of the paranasal cavity in Case 2 after the second surgery. There is no apparent evidence indicating disease recurrence.

After the surgery, we treated the patient with oral corticosteroid, nasal corticosteroid spray, and montelukast administration. Symptoms of diplopia and nasal obstruction subsided (Figures 3(a) and 3(b)), but they recurred after two years. There was exacerbation of the paranasal sinus contrast in CT images, mainly at the ethmoid bones on both sides and at the frontal sinus (Figures 3(c) and 3(d)). We also confirmed an increase of *Alternaria* in the blood and *Aspergillus*-antigen-specific IgE. We tried steroid administration and local treatments without success; we performed another surgery three years and eight months after the initial surgery. In this subsequent surgery, we used the Draf III procedure to create a single opening in the frontal sinus with a perforation of the septum, and then again removed nasal polyps and pathological mucosa in each paranasal sinus (mainly at the ethmoid sinus). In contrast with the initial surgery, we did not confirm the presence of colloidal mucus during the operation. The pathological examination of the nasal mucosa did not show the presence of fungus; only eosinophilic infiltration was evident. The patient is currently under follow-up observation and undergoing collunarium or nasal irrigation treatments. Disease recurrence has not been confirmed (Figure 4).

## 3. Case 2

**Figure 5:**
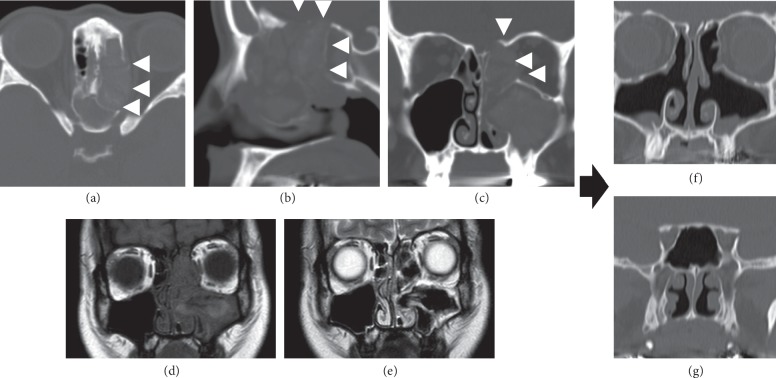
Case 2 MRI findings. (a) Computed tomography (CT) image of the paranasal cavity (axial section). (b) CT image of the paranasal cavity (sagittal section). (c) CT image of the paranasal cavity (coronal section). Soft-tissue contrasts are evident in the left maxillary sinus, the left frontal sinus, the left ethmoid sinus, and both sphenoid sinuses, along with bone thinning at the lamina papyracea and the base of the skull (white arrow). (d) T1-weighted MRI of the paranasal cavity (coronal section). (e) T2-weighted MRI of the paranasal cavity (coronal section). The paranasal sinus mucosa and nasal mucosa show isosignal intensity in the T1-weighted images, high signal intensity in the T2-weighted images, and the most of the allergic mucin show none to low signal intensity. (f, g) CT images of the paranasal cavity after the surgery (coronal section). There is no apparent evidence indicating disease recurrence.

A 34-year-old female patient visited the ear nose and throat department of the local hospital, complaining mainly of headache and dysosmia. She was diagnosed with unilateral paranasal sinusitis by CT and underwent conservative treatment, but she did not show much improvement. Since the MRI reading indicated a nasal tumor, she was referred to our hospital. There was no record of paranasal sinusitis or allergic rhinitis in her medical history. At the initial diagnosis, we confirmed the presence of a substance suggesting allergic mucin of colloidal mucus and a polyp-like lesion in the left paranasal cavity. CT images showed soft-tissue contrast filled in the left maxillary sinus, the left frontal sinus, the left ethmoid sinus, and both sphenoid sinuses, along with bone thinning at the lamina papyracea and the base of the skull (Figures 5(a)–5(c)). MRI showed isosignals in T1-weighted images of the paranasal sinus mucosa and the nasal mucosa, high signals in T2-weighted images of the paranasal sinus and the nasal mucosa, and no signals to low signals at the substance suspected to be allergic mucin (Figures 5(d) and 5(e)).

Hematological findings at the initial diagnosis included 3.5% eosinophil (213.5/*μ*L) and 550 IU/ml nonspecific IgE; as for the allergen-specific IgE, there was 3.1 pg/ml *β*-D glucan, and fungi was class 2+, *Candida* was class 2+, and *Aspergillus* was class 0. The results of a biopsy conducted on the left nasal polyp at the initial diagnosis showed only inflammatory cell infiltration or partial adhesion of filamentous fungus; we did not confirm evident malignancy.

According to these results, we eliminated the possibility of a malignant tumor and suspected AFS. We therefore performed endoscopic sinus surgery. We removed the polyps at the left nasal/paranasal cavity and cleared the pathway to each of the paranasal sinuses; for the colloidal mucus evident in the nasal/paranasal cavity, we performed a thorough irrigation using the hydrodebrider.

We examined the resected tissue samples to confirm the presence of inflammatory cell infiltration of eosinophil and neutrophil, Charcot–Leyden crystals, and altered mycelia; we also confirmed the presence of calcium oxalate crystals using a polarizing microscope. We performed short-term follow-up treatments of steroid administration and nasal irrigation; as a maintenance therapy, we continued nasal corticosteroid spray and montelukast administration. Subjective symptoms such as headache or dysosmia subsided, and CT images and visual inspection of the nasal cavity have not shown evidence of disease recurrence in the three years since the surgery (Figures 5(f) and 5(g)).

## 4. Discussion

AFS is a type of paranasal sinusitis triggered by an allergic reaction to fungus in the nasal/paranasal cavity that occurs mostly in young patients [1]. It may develop bilaterally or unilaterally; in bilateral AFS, differentiation from eosinophilic sinusitis is often challenging. Moreover, since a mucin increase in the nasal/paranasal cavity induces decalcification of the bones in the paranasal cavity, indicating bone degradation in the areas surrounding the orbital cavity or bottom of the skull just as in cases of malignant tumors, it is essential to differentiate cases of unilateral AFS from cases of malignant tumors [8]. AFS often occurs in both dry and humid regions with a warm climate [9]; it is reported mostly in India, Sudan, and Pakistan [10, 11]. The number of case reports in Japan has recently increased, suggesting the climate in Japan may be becoming favorable to AFS occurrence, a change that may be due to global warming. As for imaging characteristics, bone erosion at the orbital cavity or the bottom of the skull should be evident in CT images in 20% of AFS cases [4]; we confirmed it in both cases. These two cases satisfied all six items of the diagnostic criteria [12] put forth by the American Academy of Allergy, Asthma and Immunology (AAAAI), and we confirmed them as AFS. We conducted endoscopic sinus surgery and follow-up treatment, including oral corticosteroid medication. From the examination of the resected tissue samples, we suspected *Aspergillus* or *Alternaria* as the cause in Case 1 and confirmed evident exacerbation of paranasal sinus contrast and polyp recurrence in CT images during follow-up. Study reports indicate that AFS activity is generally intranasal, showing an eosinophil or serum-specific IgE increase in peripheral blood and a delayed increase of nonspecific IgE, while in cases of bacterial infections, an IgE increase is not evident [13]. When the disease recurred in Case 1, we confirmed contrasts located mainly at the ethmoid and frontal sinuses in CT images and polyps and mucosal hyperplasia in the nasal cavity, as well as an increase in *Aspergillus* or *Alternaria* antigen-specific IgE in the blood test, but we did not confirm a bacterial infection. We therefore suspected AFS recurrence and decided to perform another surgery. Reports indicate AFS recurrence after surgery usually in 10% to 100% [14], and follow-up steroid administration should provide preventive effects [15]. Cases of AFS recurrence reported in Japan show an increase in antigen-specific IgE or blood eosinophil in the blood test, an increase in polyps, or exacerbation of paranasal sinus contrast, and often lead to another surgery. Images taken of the tissue before the repeat surgery are usually the same as in the initial surgery, showing mucinous nasal mucus or fungi adhesion. In Case 1, however, we did not confirm the presence of fungi or mucinous nasal mucus. Only eosinophilic infiltration was evident; it was quite different from the initial surgery. With no evidence of fungi or mucinous nasal mucus, a high level of blood eosinophil, evident eosinophilic infiltration in the nasal polyp tissue (>70 HPF), and dominance in CT images in both sides of the ethmoidal sinus, we diagnosed the case as eosinophilic sinusitis according to the Japanese Epidemiological Survey of Refractory Eosinophilic Chronic Rhinosinusitis Study [16]. AFS is known to be similar to eosinophilic sinusitis, differentiated by the presence of an allergic reaction to fungi. On the other hand, one study reports that eosinophilic sinusitis is a type of disease related to eosinophilia, considered to be the systemic accommodation disorder of eosinophil at the upper and lower respiratory tract [17]. In Case 1, we did not identify fungi in the tissue below the nasal/paranasal cavity mucosa. Since a high level of eosinophilic infiltration was evident, interleukin- (IL-) 33-stimulated type 2 innate lymphoid cells by *Alternaria* and Th2 cytokines such as IL-5 or IL-13 were discharged. The influence induced eosinophilia and mucus production in the tissue, suggesting the possibility of the pathological shift to eosinophilic sinusitis due to the eosinophils increase in the tissue [18, 19]. As shown in a study reporting a case of bronchial asthma, the patient in Case 1 also had a steroid-resistant reaction during follow-up. This suggests that thymic stromal lymphopoietin produced in the respiratory tract had an effect, along with IL-33, on natural helper cells to induce a steroid-resistant reaction [20].

AAPBA, which has a pathology similar to AFS, shifts to invasive pulmonary aspergillosis due to allergic fungal asthmatics [21] or immune depression of the host during long-term steroid treatment [22]. It is considered that repeated recurrence of the disease leads to further destruction of lung tissues, and long-term steroid administration leads to a compromised defense against *Aspergillus*, resulting in a shift to the invasive disease [22]. However, pathological details of both diseases remain unknown. It is possible that AFS may shift to eosinophilic sinusitis or invasive paranasal sinus mycoses. In Case 2, on the other hand, AFS was successfully controlled without recurrence using the same treatment as in Case 1.

We used a hydrodebrider to remove the allergic mucin and pathologic mucosa, as it has been proven effective in removing clusters of fungi in the paranasal sinus mycosis or biofilms evident in the paranasal sinusitis [23]. It has also been reported that surgical operation using a hydrodebrider results in postoperative effects on cytokines [24], which suggests it is a useful device for the treatment of intractable AFS.

Changes in the disease conditions differ in each patient with AFS, and careful observation should be mandatory. Moreover, as nasal symptoms deteriorate after treatment, the possibility of a pathological shift to eosinophilic sinusitis or invasive paranasal sinus mycoses should be considered in addition to AFS recurrence. The pathology or symptoms of eosinophilic sinusitis or invasive paranasal sinus mycoses may overlap with those of AFS; close follow-up observation considering pathological changes is essential in the postoperative treatment of AFS.

## 5. Conclusion

AFS is a type of intractable paranasal sinusitis in which oral corticosteroid administration or endoscopic sinus surgery is implemented as the effective treatment method. We have encountered two cases with differing clinical observations after endoscopic sinus surgery. One showed evident disease recurrence, suggesting a pathological shift to eosinophilic sinusitis, while the other maintained a state of complete remission. This suggests that close long-term follow-up observation should be mandatory in the postoperative treatment of AFS.
